# Fatal Autochthonous Hepatitis E Induced Acute on Chronic Liver Failure Presenting with Multiorgan Failure in a Non-endemic Country: Brief Review of Intensive Care Unit Management

**DOI:** 10.7759/cureus.7490

**Published:** 2020-03-31

**Authors:** Keevan Singh, Amanda C Sang, Kevin Singh

**Affiliations:** 1 Anaesthesia and Intensive Care Unit, Department of Clinical Surgical Sciences, University of the West Indies, San Fernando, TTO; 2 Anaesthesia and Intensive Care Unit, San Fernando General Hospital, San Fernando, TTO; 3 Internal Medicine, San Fernando General Hospital, San Fernando, TTO

**Keywords:** autochthonous hepatitis e, hepatitis e virus, acute on chronic liver disease, pocus

## Abstract

Hepatitis E is usually a self-limiting disease that is considered rare in western countries. Outside of endemic regions, hepatitis E is seldom considered a cause of liver failure. We describe the first reported case of hepatitis E induced acute liver failure in the Caribbean island of Trinidad and the wider Caribbean; all traditionally considered non-endemic regions. The patient was a previously well young female who, upon investigation, was found to have radiographic signs suggesting underlying chronic liver disease. Subsequent testing yielded a positive hepatitis E immunoglobulin (Ig) M leading to the diagnose of hepatitis E induced acute on chronic liver failure. The patient’s condition quickly deteriorated following the expected pattern of multiorgan failure associated with the disease. She died after a six-day intensive care unit (ICU) stay.

## Introduction

Globally, hepatitis E is one of the most common causes of acute hepatitis [1]. Significant regional variation exists, with Asia and Africa comprising the bulk of these cases, whereas the United States and the Americas have a lower seroprevalence. There are geographic differences in hepatitis E genotypes mentioned in the literature. Genotypes 1 and 2 spread from feco-oral contamination by humans and are responsible for outbreaks in endemic regions. Subtypes 3 and 4 are spread via an animal reservoir, such as deer, pork, and wild game meat; these are responsible for sporadic outbreaks and the so-called “autochthonous” or locally acquired cases [1-2].

The majority of cases of hepatitis E are thought to be self-limiting and benign [1]. Acute liver failure (ALF) secondary to hepatitis E has a reported incidence of 28.7% in India and less than 1% in the USA [1,3]. Mortality is highest in pregnant women and those with a background of chronic liver disease, “acute on chronic liver failure (ACLF)” [1]. Hepatitis E is a common precipitant of ALF in those with a background of both known and unknown liver disease [4]. This acute on chronic liver failure, according to the Asian Pacific Association for the study of liver (APASL) definition, is associated with multiorgan failure and death in up to 50% of patients [4-5].

The prevalence of hepatitis E in the Caribbean is very low (1%) and data is lacking [6]. There is no published data on acute liver failure secondary to hepatitis E in the region. We report the first case of a fatality due to hepatitis E induced acute on chronic liver failure in the Caribbean island of Trinidad and Tobago. The potential severity of the presentation has implications for the diagnosis and treatment of acute liver failure in a region where reported cases of hepatitis E are rare. As in western countries, it appears that autochthonous hepatitis E is also present in the Caribbean. We also include a brief discussion of the intensive care unit (ICU) management, which is key for improving outcomes in a non-transplant setting.

## Case presentation

A previously well, 24-year-old Indo-Trinidadian female first presented two weeks prior to ICU admission with a short history of fever and vomiting. She received symptomatic treatment at her local general practitioner and was subsequently well enough to attend her own wedding three days later. Despite some persistent abdominal pain, she seemed her normal self during the event. The following day, she left for her honeymoon on a neighboring island. There, she experienced daily episodes of vomiting, intermittent fever, a pruritic rash, and occasional diarrhea. She was thus forced to return home three days after her departure. At home, she sought further medical attention. She was noted to be icteric and was admitted to a smaller hospital for investigation.

She had no previous blood transfusions, denied illicit drug use, consumed alcohol only occasionally, and had no tattoos. There was also no history of travel outside of the country prior to initial symptoms. Four years ago, she was hospitalized for dengue fever and pneumonia, which resolved without sequelae. No history was given of any behavioral or neurologic changes that may have indicated Wilson’s disease.

She spent five days being investigated and treated and was then transferred to our tertiary institution for further advanced management of her worsening liver function. Initial tests were negative for leptospirosis, dengue fever, hepatitis A, B, and C, and human immunodeficiency virus (HIV). Her autoimmune screen was also negative (anti-nuclear antibody (1/1000), anti-ds DNA, anti-smooth muscle antibody, and anti-mitochondrial antibody), along with her urinary pregnancy test.

**Figure 1:**
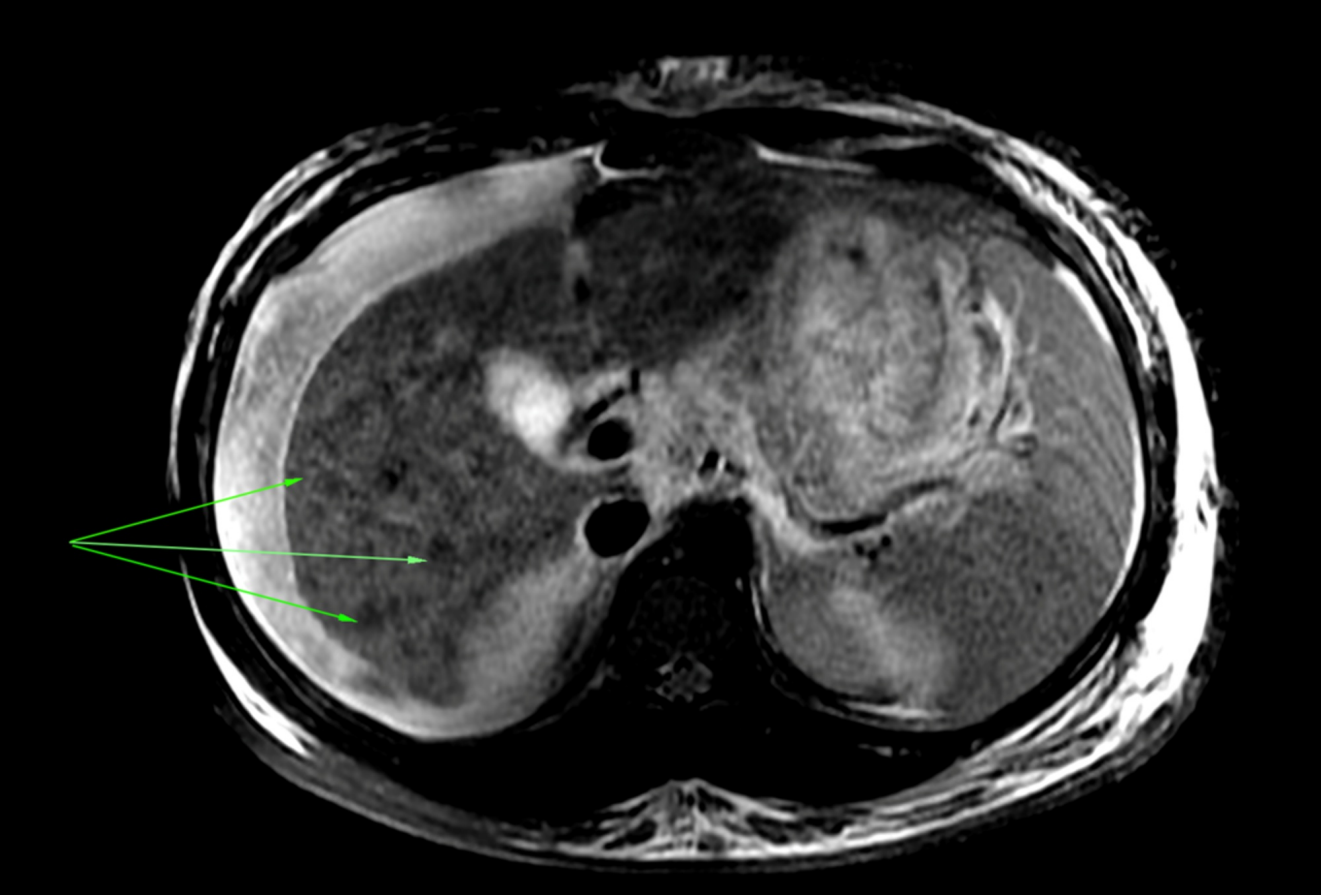
Axial T2 image demonstrating numerous regenerative nodules

**Figure 2:**
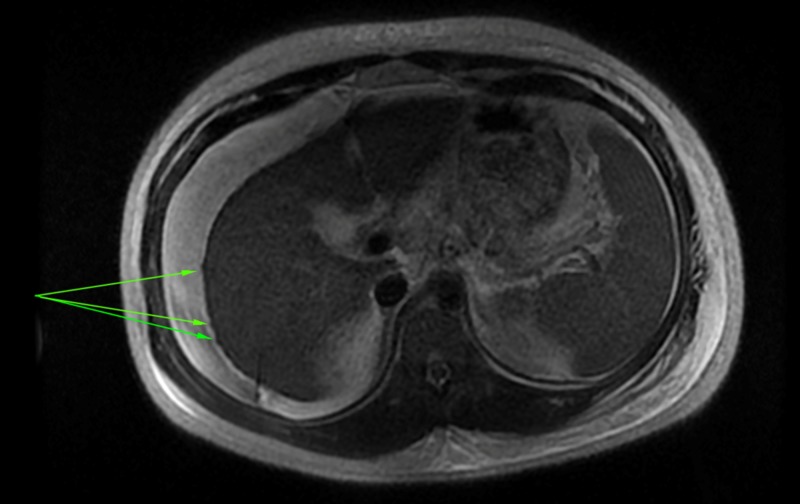
Axial T1 image showing an atrophic liver with mild sub-capsular nodularity and associated splenomegaly and ascites; features commonly seen in advanced cirrhotic disease

An abdominal ultrasound showed coarse echotexture of the liver, biliary sludge, no gallstones, no gall bladder wall thickening, and a normal common bile duct. The main portal vein measured 13 mm with no thrombosis seen and hepatopetal flow. Moderate ascites with bilateral pleural effusions were also noted. Subsequent MRI revealed a nodular liver with hepatosplenomegaly (Figures 1-2).

On examination, she was grossly icteric, mildly agitated, and had an ascitic abdomen. Her lab investigations at that time were significant for a raised white cell count - WCC (20.8 x 10^9/L), a marked rise in serum creatinine (1.5 mg/dl from 0.9 mg/dl), a transaminitis (aspartate aminotransferase (AST): 961U/L, alanine aminotransferase (ALT): 589U/L), hyperbilirubinemia (total: 41.2 mg/dl, direct: 26 mg/dl, indirect: 15 mg/dl), and her prothrombin time was reported as no coagulation. Her hemoglobin was 6.2 g/dl with no evidence of external bleeding and a negative direct and indirect Coombs test. Other significant investigations were alkaline phosphatase (ALP) of 84 U/L, gamma-glutamyl transferase (GGT) of 157 U/L, and lactate dehydrogenase (LDH) of 600 U/L.

The working diagnosis was an acute liver failure of an undetermined cause with grade I encephalopathy (West Haven). Based on her MRI and ultrasound findings (hepatosplenomegaly, nodular liver, and dilated portal vein), she was subsequently considered to also have a background of chronic liver disease. She was admitted to the ICU for further management of her liver failure. Treatment at that time consisted of intravenous (IV) piperacillin/tazobactam, oral lactulose, and fleet enemas, albumin (12.5 g IV bd), vitamin K, and IV esomeprazole in addition to general supportive ICU care. Hepatitis E serology was also requested, which showed a strongly positive IgM (4.1, Euroimmun, Lübeck, Germany).

The patient spent six days in the ICU. Persistent anuria, despite fluid resuscitation, metabolic acidosis and a rising creatinine (2.1 mg/dl), prompted the early initiation of continuous renal replacement therapy. A 50% dextrose infusion was started to treat persistent hypoglycemia. Her encephalopathy worsened over the first three days moving from grade I to grade III.

On day three of ICU, the patient had worsening oxygenation and agitation that warranted intubation, mechanical ventilation, and sedation using propofol and fentanyl infusions at 100 mg/hr and 100 ug/hr, respectively. Transaminitis and coagulation abnormalities persisted and this, together with her rapid decline and poor prospects for a liver transplant, prompted the use of N-acetyl cysteine as salvage therapy. After a brief improvement in oxygenation following intubation, further decline over the next two days required paralysis and the use of high positive end-expiratory pressure (PEEP) to improve her oxygenation. Pneumonia was suspected because of her rising white cell count (WCC). However, none was confirmed on microbiology.

On the sixth day, the patient became hypotensive and a noradrenaline infusion was started at .8 ug/kg/min. On examination, she had bounding pulses and warm peripheries suggestive of vasodilatory shock. The hypotension persisted and she was noted to have bilateral dilated pupils associated with bradycardia of 40 to 45 beats per minute as seen on a real-time cardiac monitor and a low mean arterial pressure (MAP; <50).

**Figure 3:**
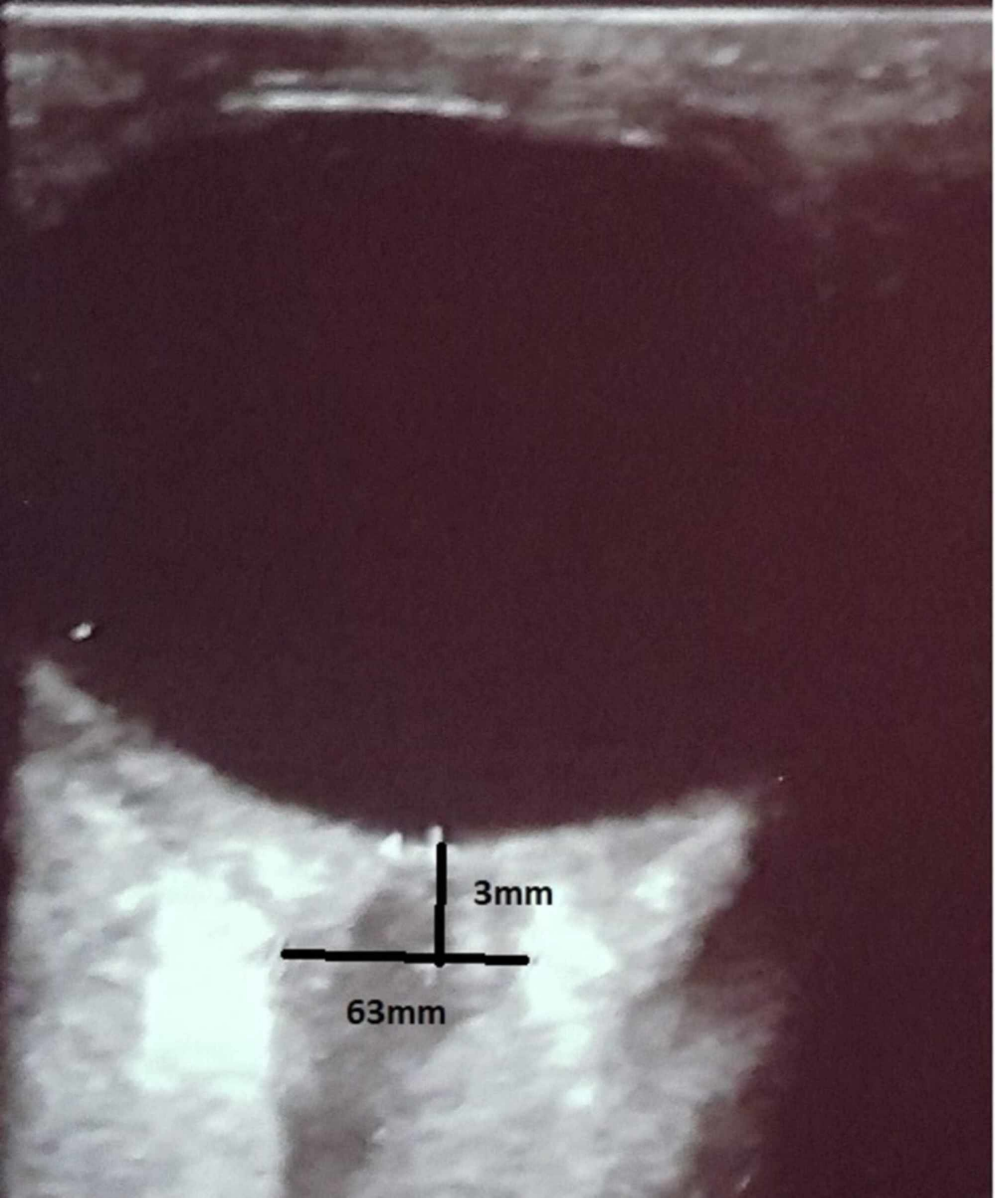
The patient’s enlarged ONSD measured 3 mm posterior to the retina with a high-frequency ultrasound probe ONSD: optic nerve sheath diameter

Using point-of-care ultrasound, cardiac contractility was noted to be preserved, with a full inferior vena cava with minimal respiratory variation, and lung sliding was seen bilaterally with B-lines noted over the lung fields. Her optic nerve sheath diameter measured 63 mm, using a high-frequency ultrasound probe (Figure 3).

This together with her clinical state likely indicated raised intracranial pressure (ICP). The noradrenaline dose was increased to 1 ug/kg/min and an adrenaline infusion added in an attempt to increase her mean arterial pressure (MAP) to improve her cerebral perfusion pressure in the setting of a raised ICP. Respiratory rate was also increased to attempt to reduce her ICP while awaiting stabilization for CT brain. However, despite escalating inotropic and vasopressor requirements, her blood pressure and heart rate continued to fall, leading to an eventual fatal cardiac arrest shortly after her dilated pupils were noted.

## Discussion

Acute on chronic liver failure is a relatively new clinical condition that is distinct from the related decompensated cirrhosis and ALF. Consensus guidelines have defined it as “an acute hepatic insult manifesting as jaundice and coagulopathy, complicated within 4 weeks by clinical ascites and/or encephalopathy in a patient with previously diagnosed or undiagnosed chronic liver disease or cirrhosis, and is associated with a high 28-day mortality" [5].

ACLF is associated with a transplant-free survival of up to 50% [4]. Mortality rates for hepatitis E induced ACLF in endemic regions range from 34% up to 67% [7]. A seven-day window period is described whereby specific treatment of the underlying cause of the acute deterioration and supportive liver treatment can lead to the reversal of the ACLF [4-5]. Hence, early diagnosis and treatment is key.

The immune response plays a key role in ACLF, with systemic inflammatory response syndrome (SIRS) and a sepsis-like presentation being common in ACLF [5]. Doubt exists whether this sepsis-like response is the cause or a consequence of liver failure and, in many cases, no source of infection is identified [4]. Consequently, organ dysfunction is reported to occur in 40% of patients with ACLF and persistence of the SIRS response during the seven-day window period is associated with high mortality [4]. This persistent immune dysregulation is a key feature of ACLF progressing from SIRS to multiorgan failure, with the number of organs failing to be significantly correlated to mortality [8].

In a series of ACLF in India by Garg et al., the majority of patients had no stigmata of chronic liver disease and the cause of the chronic liver failure could not be found in 24% of patients [8]. Consensus guidelines have also acknowledged that many patients with ACLF present without any prior assessment of liver disease [5]. Likewise, the diagnosis in our patient was only possible using radiographic criteria (splenomegaly, nodular liver) similar to that used by Radha Krishna et al. [9].

The patient’s clinical course was one of progressive organ dysfunction characteristic of ACLF and as described by Sarin et al., with her initial sequential organ failure assessment score (SOFA) score being 8 and increasing to 17 on her last day [4]. A variety of prognostic scoring systems has been suggested for use in ACLF, however, none have been prospectively validated. These systems include SOFA, chronic liver failure-sequential organ failure assessment (CLIF-SOFA), and model end-stage liver disease (MELD) scores [5]. Generally, worsening organ failure is associated with worse prognosis [8]. Higher-grade encephalopathy, hyponatremia, and renal failure have also been associated with a poorer prognosis in ACLF [9].

Care for patients with ACLF, can be classified as general supportive care, transplantation, and liver support therapies as well as a specific treatment for the acute etiology. Using the above-mentioned scoring systems, the case described may have benefited from early liver transplantation. However, her rapid decline with multiorgan failure and the lack of any transplant centers in close proximity precluded this. This is a common challenge in ACLF, where its progressive multiorgan failure and sepsis limits the role of transplantation. Hence, general supportive critical care and liver-specific treatment is key in these patient groups. In ACLF, liver support with artificial liver support systems may provide bridging therapy until a donor is available or the patient is stable enough to undergo such procedure [4].

Improvements in critical care have allowed for transplant-free survival of up to 60% in acute liver failure [4]. ICU management in ACLF focuses on general organ support that allows time for hepatocyte regeneration or stabilization for liver transplantation. Specific ICU treatment involves the use of vasopressors for the treatment of shock, volume expansion, antibiotics, sedation, mechanical ventilation, and the use of continuous renal replacement therapy [10]. Noradrenaline is usually the vasopressor of choice however, terlipressin, which is also useful in acute kidney disease secondary to the hepatorenal syndrome, can reduce noradrenaline requirements [7,11]. Mechanical ventilation, sedation with propofol, and specific ICP lowering therapies, such as mannitol, may also be needed to reduce raised ICP associated with high-grade encephalopathy [12].

Point of care ultrasound (POCUS) use has become commonplace in most ICUs since they allow advance monitoring and real-time decision-making [13]. This modality can prove to be particularly useful in the care of the patient with ACLF with its known rapid progression. In our case, POCUS allowed us to rule out other treatable causes of shock and detect a potentially raised ICP prior to the patient’s demise. Raised ICP is transmitted to the subarachnoid space, which surrounds the optic nerve, causing optic nerve expansion and, as such, is translated as an increase in optic nerve diameter [14]. Recent studies confirm that ONSD measurement correlates with ICP measurements; with one study quoting ONSD measurement having 94.4% sensitivity and 95.2% specificity in traumatic brain injury patients [14-15]. Given the potential complications of invasive ICP monitoring in coagulopathic patients and its varied availability in different regions, this may warrant a prospective trial on the use of optic nerve ultrasound in acute liver failure.

Specific antiviral therapies have also been advocated for viral causes of ACLF. However, no specific antiviral therapy has been advocated for the treatment of the hepatitis E virus. A few reports have shown positive results with peginterferon and ribavirin in treating the hepatitis E virus and this may prove to be promising future therapy in ACLF [1,7]. Although N-acetylcysteine (NAC) is generally not recommended for use in non-acetaminophen liver failure, meta-analyses have documented its ability to prolong survival and its safety in ALF [16]. Thus, NAC can serve as a pharmacologic bridging therapy in these patients.

## Conclusions

Although the hepatitis E virus is considered relatively benign, patients with a known and unknown background of chronic liver disease can present with a devastating ACLF and multiorgan failure. Hepatitis E is not only confined to endemic regions but exists in many countries in an indigenous or autochthonous form. Clinicians need to be aware of this presentation and the subsequent need for critical care admission and possible liver transplantation, as there is usually only a narrow therapeutic window in which to act.
